# Verrucous psoriasis and current treatments: a review of the literature

**DOI:** 10.1007/s00403-025-04085-2

**Published:** 2025-03-19

**Authors:** S. Minhaj Rahman, Omar Alani, Aasheen Qadri, Fahad Ahmed, Adel Haque

**Affiliations:** 1 Department of Dermatology, University of Rochester School of Medicine & Dentistry, Rochester, NY USA; 2 Department of Dermatology, Icahn School of Medicine at Mount Sinai, New York, NY USA; 3 Department of Dermatology, University of Maryland School of Medicine, Baltimore, MD USA; 4 Department of Dermatology, University of Miami/Jackson Health System, Miami, FL USA; 5 Dermatology Partners, Macungie, PA USA

**Keywords:** Verrucous psoriasis, Psoriasis treatment, Topical therapies, Systemic therapies, Physical treatments

Verrucous psoriasis (VP) is a distinct subtype of psoriasis (PsO) characterized by hyperkeratotic lesions on an erythematous base [1]. Given its rare occurrence and notorious treatment resistance, no consensus exists regarding first-line or subsequent treatment regimens, leading to frequent attempts with off-label therapies. This review aims to consolidate current literature regarding efficacious treatment regimens, to better guide clinicians when managing this uncommon condition.

A comprehensive systematic literature search for peer-reviewed articles in English was performed using PubMed, SCOPUS, and EMBASE databases, from inception to October 2024. Search terms included variations of “verrucous psoriasis” and treatment types. Two authors (SMR & AQ) searched for eligible literature that discussed the treatment of VP. Data on study design, patient demographics, dermatologic and non-dermatologic co-morbidities, treatment agents, dosage, frequency, and results of treatments were extracted.

Of the 707 articles reviewed, 20 met the inclusion and exclusion criteria, comprising 19 individual case reports and one letter proposing a diagnosis of VP in a previously published case report (Fig. 1). Of the included 20 patients with VP, the mean age was 56.4 (SD 21.7) years and the majority were male (75%). Three patients (15%) had mechanical removal of plaques through surgical excision, ablation, or forceps, and reported no lesion improvement. One in three patients was successfully treated with cryotherapy with no recurrence after 6 months [2].

Most patients (75%, *n* = 15) were treated with steroid and adjuvant combination therapy leading to significant resolution of VP plaques for four patients (26.7%), with only one patient experiencing post-inflammatory hyperpigmentation. However, nine cases (60%) reported no improvement (mean treatment 2–24 weeks). Oral apremilast displayed a 50% reduction in lesion size and flattening and regression of plaques in two patients [3, 4]. Etretinate or acitretin demonstrated improvement for four (44.4%) out of nine patients resulting in gradually decreased verrucous lesions, with one patient developing alopecia on acitretin, a noted adverse effect [5]. A summary of all treatment efficacy is reported in Table 1.

Three patients (15%) received oral or intramuscular methotrexate with adjuvant acitretin, two of which demonstrated some lesional improvement (66.7%). Biologics and systemic therapies were trialed nine times across five patients. Significant improvement was noted with adalimumab in one of three patients (33%), ixekizumab with adjuvant topical steroids (*n* = 1) and ustekinumab (*n* = 1), partial improvement in one of two patients treated with etanercept, and no improvement with infliximab (*n* = 1). Overall, off-label biologic therapy can vary greatly, and additional research is required.

Although there was some improvement to traditional treatment approaches of steroids with adjuvant therapy, alternative regimens such as apremilast, vitamin A derivatives, methotrexate, and off-label biologics have also displayed great efficacy. The efficacy of biologic agents in managing VP may indicate a shared inflammatory pathogenesis with PsO involving Th1 and Th17 cells, targeting keratinocyte hyperproliferation. However, no single regimen proved universally effective, highlighting the need for further research to identify more effective treatments. This study is limited by the lack of clinical trials or cohort studies in the literature, likely due to the rarity of the disease.

**Fig. 1:**
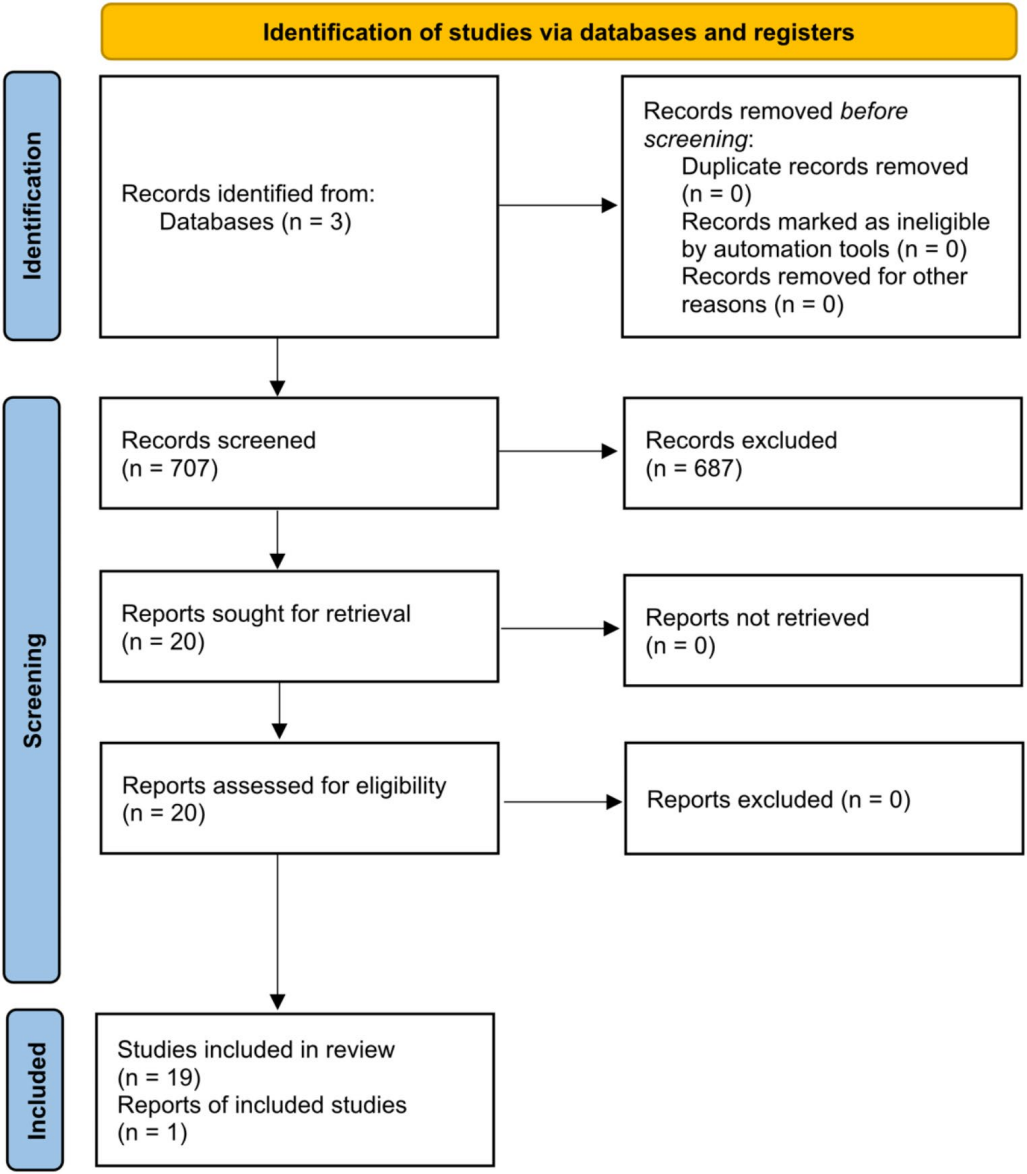
PRISMA flow diagram of study selection process

**Table 1:** Summary of therapeutic approaches and outcomes for verrucous psoriasis

Treatment Agent	Study Author(s) and Year Published	Study Design	Age/Gender	Previous Treatment (outcome)	Dosing Regimen	Results	Adverse Events
Apremilast	Xenopoulou 2023	CR	64 F	- Betamethasone (ineffective) - Narrowband UV-B (ineffective)	Day 1: 10 mg AM Day 2: 10 mg AM and PM Day 3: 10 mg AM 20 mg PM Day 4: 20 mg AM and PM Day 5: 20 mg AM and 30 mg PM Day 6 and after: 30 mg AM and PM	Within 28 days, 50% reduction in lesion size, decreased in severity	Mild gastrointestinal upset which self-resolved
	Okazaki 2019	CR	67 M	N/A		Within 9 months, trunk plaque psoriasis flattened and leg verrucous psoriasis regressed	Yes, Not Specified
Adalimumab	Maejima 2012	CR	55 M	- Oral cyclosporine for a year (partially effective)	40 mg every other week (5 months)	Decreased PASI from 16.2 to 3.7	N/A
Ixekizumab	Sherkin 2022	CR	14 M	- Adalimumab (ineffective) - Acitretin (ineffective)	N/A; used with topical corticosteroids (6 months)	BSA from > 50–2%	N/A
Ustekinumab	Curtis 2012	CR	46 M	- Tx regimen of topical keratolytics, high-potency topical steroids, and acitretin 50 mg daily (ineffective) - MTX 15 mg/weekly (ineffective) - Etanercept 50 mg weekly (ineffective) - Adalimumab 40 mg every other week (ineffective) - Infliximab 5 mg/kg every 6–8 weeks (ineffective)	45 mg/12 weeks (9 months)	Moderate Improvement	N/A
Methotrexate	Shivers 2019	CR	76 F	- Topical triamcinolone ointment (ineffective) - Oral antibiotics (ineffective)	Tx 1: Oral; 20 mg/week (15 months) then 10 mg/week with acitretin 25 mg/day (N/A) Tx 2: Followed with compounded corticosteroid ointment containing liquor carbonis detergens, salicylic acid, and fluocinonide ointment	Tx 1: partial plaque improvement Tx2: Minor additional benefit	Mild cheilitis
	Akshaya 2022	CR	57 M	N/A	Intramuscular injection; 12.5 mg/week along with acitretin 25 mg/day and topical high potent steroid with keratolytic agent (8 weeks)	Lesions Improved	N/A
Vitamin A Derivatives	Okuyama 2006	CR	60 M	- Removed lesioned scales mechanically and applied topical vitamin D3, corticosteroids, and topical PUVA (ineffective)	Oral etretinate; 30 mg/day (2 months)	Psoriatic lesions became flat, leaving mild erythema and pigmented macules	N/A
	Wakamatsu 2010	CR	39 M	- Cryotherapy (ineffective)	Oral etretinate; 30 mg⁄day with topical vitamin D and compression bandaging (3 weeks)	verrucous lesions gradually flattened, decreased PASI from 28.8 to 20.8	N/A
	Larsen 2007	CR	42 M	- Topical keratolytic agents (ineffective) - Cryotherapy (ineffective) - Topical contact sensitizer of dinitrochlorobenzene (ineffective)	Acitretin 50 mg/day (6 months) tapered to 10 mg/day which caused relapse. Dose was increased to 35 mg/day	Complete resolution	N/A
	Iinuma 2018	CR	72 M	- Etretinate 10 mg/day (N/A)	Etretinate 20 mg/day with mechanical removing of scales, and topical vitamin D3 and corticosteroids (5 weeks)	Lesions gradually flattened	N/A
Steroid + Adjuvant Combination Therapy	Erkek 2001	CR	22 F	Intermittent topical corticosteroids and coal tar derivatives (partially effective with recurrence)	Topical; moderate potency with crude coal tar 5% (15 days)	Lesions healed	Post-inflammatory hyperpigmentation
	Monroe 2011	CR	84 F	- Etanercept (partially effective) - UV-B phototherapy (N/A) - Acitretin 25 mg/day (N/A; had AE) - Topical steroids: triamcinolone, halobetasol, clobetasol, and betamethasone/calcipotriene combination ointments (ineffective)	Topical fluocinonide and keratolytic agents, urea and salicylic acid (N/A)	Resolution of plaques on extremities and chest, but truncal plaque developed	N/A
	Su 2023	CR	95 M	- Topical miconazole cream 2x daily (partially effective)	Topical monotherapy with betamethasone dipropionate and calcipotriol ointment 1x daily (20 weeks)	Significantly decreased verrucous nodules and near complete resolution of erythematous patches	N/A
	Sergeyenko 2017	CR	53 F	- Salicylic acid (ineffective)	Topical tazarotene cream in the morning and clobetasol propionate cream at night under occlusion (N/A)	N/A	N/A
	Sezer 2015	CR	26 M	N/A	Topical salicylic acid 5% and corticosteroid ointment including betamethasone valerate (N/A)	Marked generalized lesional regression	N/A
Surgical	Sanyal 2021	CR	80 M	N/A	Triamcinolone acetonide ointment, with selective periodic surgical excision (N/A)	N/A	N/A
	Garvie 2019	CR	81 M	- Phototherapy, potent topical steroids, and several biologics (ineffective)	Staged Surgical Excision (N/A)	Graft break-down and poor wound healing	painful plantar pustulosis
Cryotherapy	Nakamura 1994	CR	60 M	- Topical corticosteroids (ineffective) -− 2 mg/g 1α,24R-dihydroxy cholecalciferol ointment (ineffective) -− 2% coal tar ointment (ineffective) PUVA bath (ineffective)	Cryotherapy (N/A)	Lesions resolved with no recurrence (6 month follow up)	N/A
Other	Scavo 2004	CR	44 M	N/A	Cessation of (IFN)-α treatment with emollients and systemic antihistamine (3 months)	Complete resolution of verrucous lesions	N/A

CR– Case report; F– Female; M- male; UV-B– Ultraviolet B; mg– milligram; AM– morning; PM- evening; PASI– psoriasis.Area and severity index; BSA– Body surface area; N/A– not applicable or not specified; Tx– Treatment; MTX– Methotrexate; PUVA - Psoralen plus ultraviolet A; IFN- α– Interferon alpha

## Data Availability

No datasets were generated or analysed during the current study.
